# Neuroinflammation: targeting microglia for neuroprotection and repair after spinal cord injury

**DOI:** 10.3389/fimmu.2025.1670650

**Published:** 2025-10-06

**Authors:** Roberta Ramos Cavalcanti, Fernanda Martins Almeida, Ana Maria Blanco Martinez, Camila Marques Freria

**Affiliations:** ^1^ Pathological Anatomy Program at Clementino Fraga Filho University Hospital, Federal University of Rio de Janeiro, Rio de Janeiro, RJ, Brazil; ^2^ Center for Neural Repair, Dept. of Neurosciences, University of California - San Diego, San Diego, CA, United States

**Keywords:** spinal cord injury, microglia, macrophages, neuroinflammation, blood brain barrier

## Abstract

Neuroinflammation is a tightly regulated process essential for central nervous system (CNS) homeostasis, debris clearance, and defense against pathogens. Microglia, the resident immune cells of the CNS, are central to this response, supporting plasticity and repair under normal conditions. Following spinal cord injury (SCI), however, this response becomes amplified and dysregulated. Early microglial activation can be protective, but prolonged activation drives the release of pro-inflammatory and cytotoxic mediators that exacerbate secondary injury and hinder repair. Microglia also engage in complex crosstalk with astrocytes, oligodendrocytes, neurons, and infiltrating immune cells, orchestrating both protective and damaging processes. This dual and dynamic nature underscores their importance as both targets and modulators in SCI therapies. This review aims to examine the roles of microglia in SCI, summarizes SCI pathology, the specific roles of microglia and macrophages, and outlines translational efforts to modulate their activation, while also highlighting the barriers to clinical application. Evidence from preclinical studies and emerging therapeutic strategies, including pharmacological, cell-based, and exosome-based interventions, demonstrates the potential to reduce harmful inflammation, promote neuroprotection, and support functional recovery. Despite these advances, clinical translation remains limited, constrained by the heterogeneity of microglial responses, narrow therapeutic windows, and patient-specific variability. These challenges often lead to modest or inconsistent clinical outcomes. Future strategies will require precision, multi-targeted approaches that integrate microglial modulation with the preservation of the blood–brain barrier (BBB) and the regulation of peripheral immune infiltration. Harnessing the regenerative potential of microglia, guided by biomarker-based patient stratification and a deeper understanding of their dynamic roles, offers the most promising path toward meaningful recovery after SCI.

## 1 Introduction

Under normal, healthy conditions, neuroinflammation is a vital and protective process essential for maintaining Central Nervous System (CNS) homeostasis. This tightly regulated immune response is primarily orchestrated by resident glial cells, particularly microglia, the CNS’s innate immune cells, and astrocytes (1, 2). In this physiological state, these cells work synergistically to support neuronal function, modulate synaptic activity, and efficiently clear cellular debris and pathogens (3, 4). This moderate level of inflammatory signaling is indispensable for healthy brain function and neural adaptability.

Following SCI, neuroinflammation is triggered by both cellular and molecular mechanisms aimed at containing the injury and promoting tissue repair (5–7). The acute inflammatory response involves the activation of resident CNS glial cells, including microglia and astrocytes, as well as the recruitment of peripheral immune cells to the injury site. Microglia, the primary innate immune cells of the CNS, are among the first responders to SCI. Their activation results in the release of pro-inflammatory cytokines, such as tumor necrosis factor-alpha (TNF-α) and interleukin-1 beta (IL-1β), which in turn recruit peripheral immune cells like macrophages and neutrophils (8). This early phase neuroinflammation plays a vital role in clearing cellular debris (9), containing the damage (5), and promoting tissue repair through the release of neurotrophic factors that support neuronal survival and axonal growth (10). While beneficial in the acute phase, prolonged neuroinflammation can transition to a chronic state that exacerbates tissue injury, impedes repair processes, and hinders functional recovery.

Chronic neuroinflammation is characterized by sustained activation of microglia, astrocytes, and infiltrating peripheral immune cells. Over time, the continued release of pro-inflammatory cytokines and chemokines disrupts the delicate balance of the CNS, contributing to secondary injury. One of the key outcomes of chronic inflammation is the breakdown of the blood-brain barrier (BBB), leading to a “leaky brain” state. This increased BBB permeability allows for the infiltration of peripheral immune cells, such as macrophages, neutrophils, and T-lymphocytes, as well as serum proteins, into the CNS (11). These infiltrating immune cells exacerbate the inflammatory response, further increasing neuronal damage (12–14).

Recent studies highlight the significant role of the local microenvironment in shaping the neuroinflammatory response, with microglia and peripheral immune cells acting in concert to amplify the inflammatory cascade. The release of pro-inflammatory cytokines such as IL-1β, interleukin-6 (IL-6), interleukin-17 (IL-17), and TNF-α, in combination with chemokines like CCL2 and CXCL10, contributes to a self-perpetuating cycle of inflammation (8). These mediators not only amplify neuronal damage through excitotoxicity but also recruit additional immune cells, creating a feedback loop that exacerbates injury. This dysregulated response, where the immune system fails to transition into a resolution phase, is a central driver of SCI pathophysiology. As a result, while microglia play an essential role in normal CNS function (7, 8, 14), their uncontrolled activation after SCI significantly contributes to secondary injury and creates an environment hostile to recovery.

Given the central role of neuroinflammation in SCI pathophysiology, therapeutic strategies aimed at modulating this response have become a major focus of research. Traditionally, anti-inflammatory therapies have aimed to suppress the inflammatory response to prevent further tissue damage. However, this approach has limitations, as inflammation is necessary for initial repair and tissue remodeling. Recent advances have focused on reprogramming or guiding microglia towards a beneficial, pro-regenerative phenotype that supports recovery without exacerbating injury.

Several novel therapeutic approaches have emerged, including the use of small molecules, gene therapies, and biologics aimed at selectively modulating microglial activity and promoting the resolution of inflammation. Additionally, strategies targeting the BBB, such as the use of stabilizing agents or immune cell-targeting therapies, hold promise in preventing excessive immune cell infiltration and promoting tissue repair.

This review examines the coordinated interactions between microglia, neurons, other glial cells, peripheral immune cells infiltrating through a compromised blood–brain barrier (BBB), and the extracellular matrix in the context of spinal cord injury (SCI). These interactions, mediated by direct cell signaling, crosstalk, and the regulation of cytokines, neurotrophins, and neurotransmitters, are dynamic and context-dependent. They play a central role in shaping the microglial immune response, which in turn influences the progression of injury and the potential for repair. By identifying key cellular and molecular players, particularly the diverse phenotypes of microglia and their responses to environmental cues, this review highlights potential therapeutic targets and innovative strategies to reduce CNS damage and improve clinical outcomes.

## 2 Physiological neuroinflammation, normal brain function, and plasticity

Under normal physiological conditions, microglia play a vital role in maintaining brain homeostasis (15, 16). One of their primary functions is phagocytosis—the engulfment and degradation of cellular debris, including apoptotic (dying) cells (15, 17). In the healthy adult brain, microglia act as the main phagocytic cells, efficiently clearing apoptotic debris. This clearance is essential to prevent unnecessary inflammation, as the timely removal of dying cells limits the release of proinflammatory cytokines, which could otherwise become neurotoxic (18).

**Figure 1:**
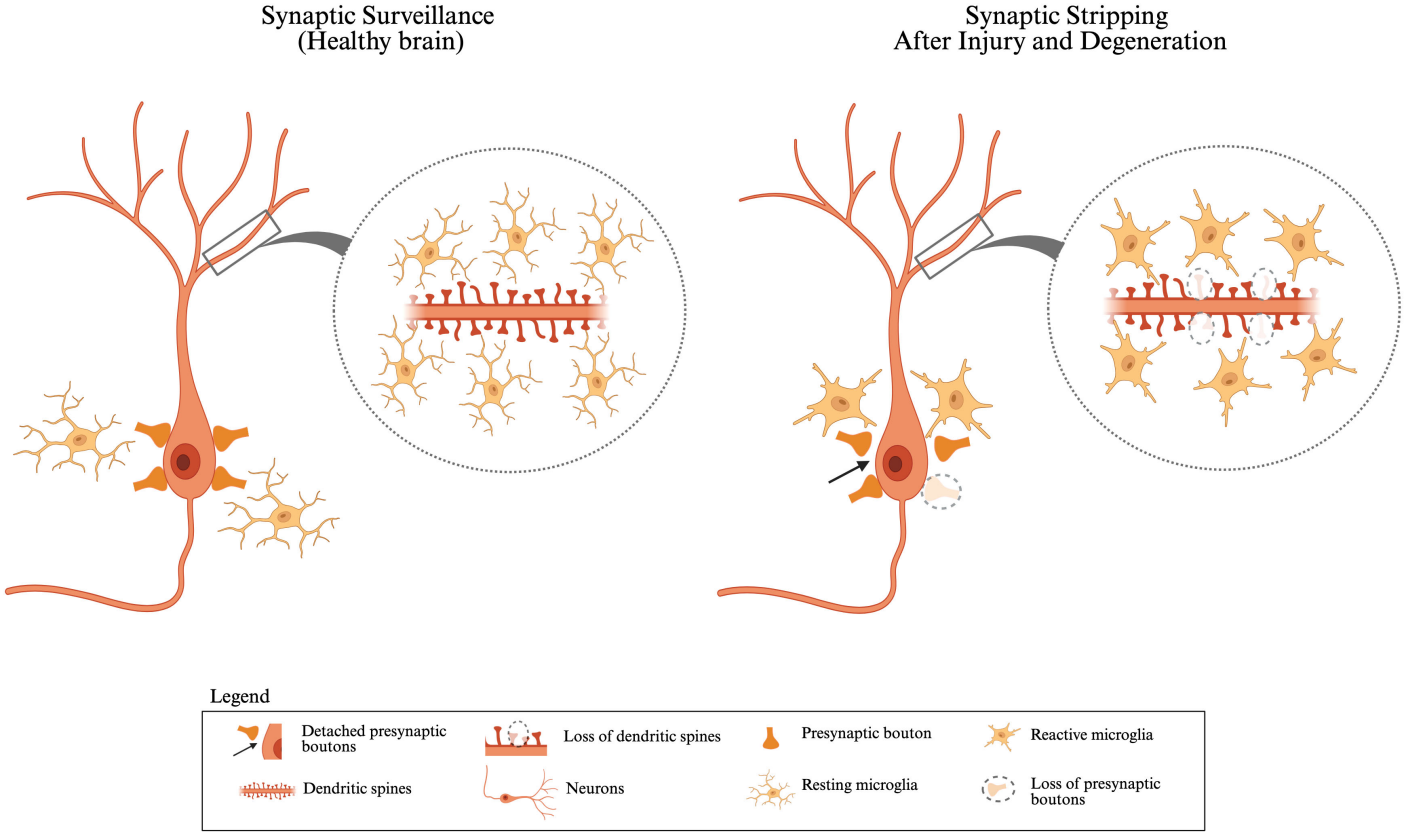
Synaptic surveillance in healthy brain *vs*. synaptic stripping after injury and degeneration: In a healthy brain, resting microglia closely monitor and interact with neurons and maintain synaptic homeostasis. Neurons display intact dendritic spines, and presynaptic boutons are stably connected, indicating normal synaptic surveillance. During development, microglia can phagocytose “weak” or unnecessary synapses to regulate synapse numbers. In response to injury or neurodegeneration, microglia become reactive and can alter synaptic function by phagocytosing damaged or dysfunctional synapses. Reactive microglia surround the neuron and facilitate the detachment of presynaptic boutons (highlighted by arrows), along with the loss of dendritic spines and presynaptic receptors (shown by dashed circles). This figure was created with BioRender.com.

Beyond debris clearance, microglia continuously monitor the brain environment and interact with synapses (17, 19–22). During brain development, they contribute to synaptic pruning, a process that eliminates excess or inappropriate neuronal connections to support the formation of well-organized neural circuits (23). Disruptions in synaptic pruning during childhood brain development, whether excessive or insufficient, have been linked to neurodevelopmental disorders such as autism, epilepsy, and schizophrenia (24). This synaptic remodeling continues into adulthood as part of the brain’s capacity for plasticity, forming a variety of microglial activity from early development through maturity (20, 21). Over the past decade, two-photon microscopy has revealed that microglia continuously survey their surrounding microenvironment and synapses through dynamic, actin-mediated process movements. These processes make transient contacts with both pre- and postsynaptic structures, allowing microglia to monitor and respond to changes in synaptic activity (17, 25). Importantly, this normal synaptic pruning is distinct from the pathological overactivation of microglia that can lead to the phagocytosis of viable neurons that is a detrimental process associated with disrupted neuron–microglia signaling (26) (**Figure 1**).

In a healthy state, microglia not only maintain homeostasis but also contribute to ongoing synaptic remodeling by sensing synaptic activity and forming transient synaptic contacts. Studies in mice lacking CX3CR1, a chemokine receptor expressed by microglia that binds to the neuronal ligand CX3CL1 (fractalkine), have demonstrated impaired synaptic development. These CX3CR1-deficient mice show a reduced number of microglia during the postnatal period and transient defects in synaptic connectivity and plasticity, particularly in the hippocampus (27). While prolonged microglial contact with synapses is often associated with disease states (28), growing evidence indicates that microglia also contribute to healthy synaptic remodeling and plasticity under normal conditions (16, 17, 29). For instance, in the somatosensory cortex of young mice, microglia make contacts with both pre-synaptic boutons and postsynaptic spines synapses with a frequency of about 1 contact/hour (17).

Synaptic pruning, which fine-tunes neural circuits in response to experience and learning, continues into adulthood. This process involves the strengthening of functionally relevant synapses and removing redundant ones, optimizing neural communication for efficient and adaptive information processing (30) (**Figure 1**). These findings highlight the essential role of microglia in shaping and optimizing synaptic networks that support cognitive function.

## 3 Pathophysiology of neuroinflammation in SCI

Following SCI, the intricate neuroinflammatory response to the initial contusion and/or compression, bone fracture, hemorrhage, and membrane disruption is primarily orchestrated by resident microglia and neutrophils, subsequently joined by astrocytes and infiltrating monocytes (6, 31–35). These initial responders initiate widespread inflammatory cascades through the recruitment of additional immune cells and complex cellular signaling pathways. Peripheral immune cells, including neutrophils, monocytes, natural killer (NK) cells, dendritic cells, T-cells, and even B-cells, play crucial roles in this multifaceted immune response (11, 36).

Neutrophils, as key participants in the immune response, actively engulf lesion debris and contribute to the breakdown of the blood-brain barrier (BBB) through the release of free radicals, proteolytic enzymes, and matrix metalloproteinases (37). Furthermore, their migration into the CNS is associated with the release of interleukin-17 (IL-17), which contributes to cytotoxicity and further BBB disruption (37). Monocytes, a type of white blood cell, differentiate into monocyte-derived macrophages (MDM) and monocyte-derived dendritic cells (38). These infiltrating blood-borne monocytes gain access to the CNS via a compromised or even intact BBB. They are critically involved in demyelination processes within the CNS and significantly contribute to the secretion of pro and anti-inflammatory factors that regulate pathological events following SCI (6, 39, 40).

**Figure 2:**
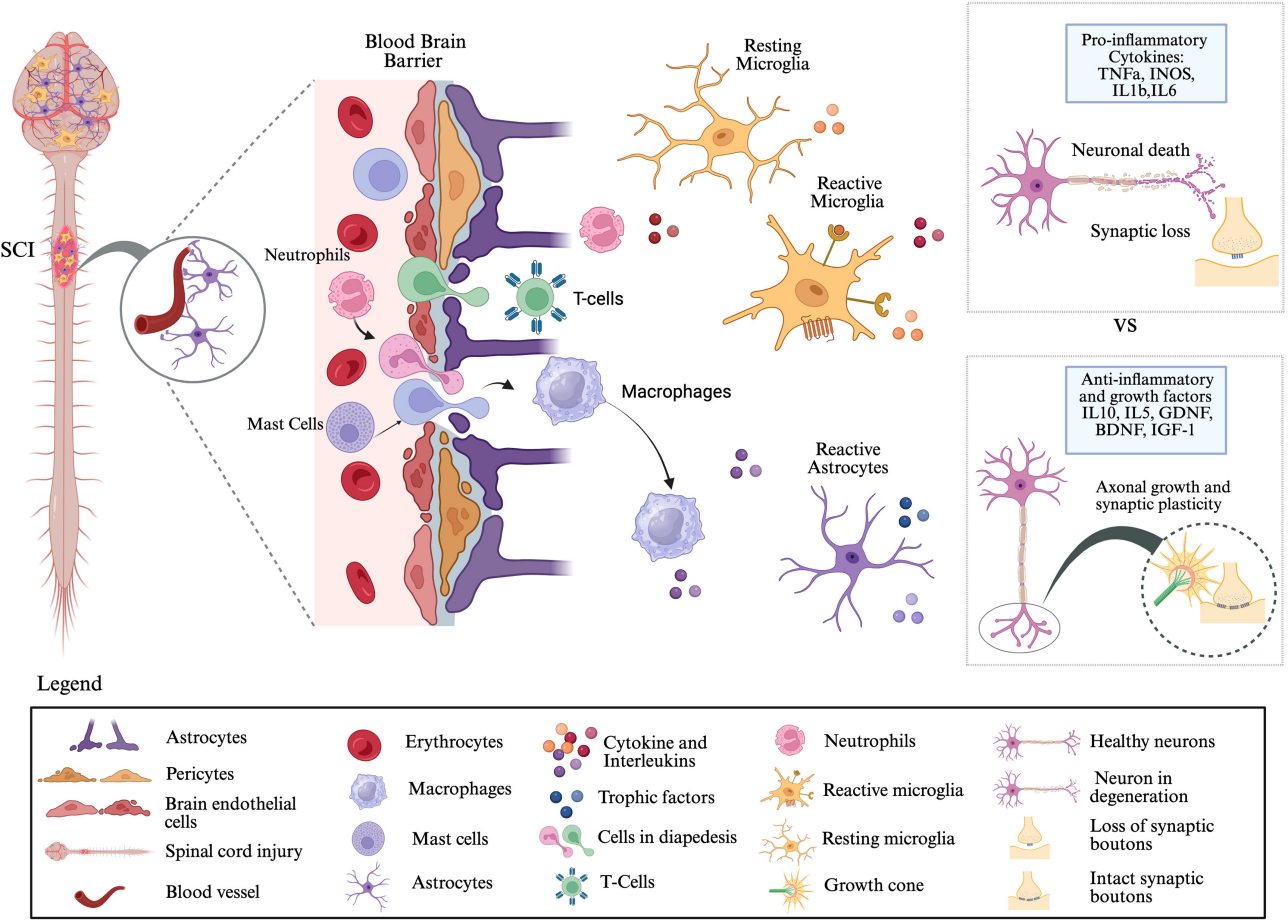
Neuroinflammatory responses following spinal cord injury: Traumatic SCI disrupts the blood–brain barrier (BBB), which is normally maintained by astrocytes and pericytes. Following injury, BBB integrity is compromised, creating gaps that allow infiltration of peripheral immune cells, including neutrophils, T cells, and macrophages, into the central nervous system (CNS) parenchyma. These infiltrating cells, together with resident mast cells, release cytokines and interleukins that activate microglia and astrocytes. Microglia, the primary immune responders in the CNS, undergo a transition from resting to reactive states and, along with reactive astrocytes, secrete both pro-inflammatory mediators (e.g., TNFα, iNOS, IL-1β, IL-6) and anti-inflammatory/growth factors (e.g., IL-10, IL-5, GDNF, BDNF, IGF-1). The balance between these opposing signals determines the pathological outcome: pro-inflammatory signaling promotes neuronal death and synaptic loss, whereas anti-inflammatory and trophic signaling supports axonal regeneration, synaptic plasticity, and potential functional recovery. This figure was created with BioRender.com.

Within hours of injury, resident microglia, astrocytes, and infiltrating leukocytes begin to secrete a broad array of pro-inflammatory cytokines, including TNF-α (41), IL-1β, and IL-6 (42), which amplify the inflammatory response and promote further immune cell infiltration (37, 38). Clinical data from human cerebrospinal fluid (CSF) samples show that, within 24 hours post-injury, levels of IL-6, IL-8, and monocyte chemoattractant protein-1 (MCP-1), along with structural injury markers such as glial fibrillary acidic protein (GFAP), tau, and S100β, are significantly elevated in patients with severe SCI (43). Similarly, in experimental rat models, levels of TNF-α, IL-2, IL-10, IL-17α, and interferon-gamma (IFN-γ) increase significantly in the CSF within 6 hours post-injury, and serum concentrations of TNF-α, IL-1β, and IL-6 remain elevated for at least a week after SCI (44). Compensating this pro-inflammatory response, anti-inflammatory cytokines such as interleukin-10 (IL-10) and transforming growth factor-beta (TGF-β) play essential roles in limiting immune cell cytotoxicity, dampening inflammation, and promoting neuroprotection (45, 46) (**Figure 2**). The interplay between these opposing cytokine signals determines the trajectory of the injury response and functional recovery.

Chemokines such as CCL2 (MCP-1), CXCL10 (IP-10), and CXCL1 are also key mediators in the recruitment and spatial organization of immune cells at the injury site (47). For instance, CCL2 attracts monocytes and macrophages that are critical for phagocytosis and tissue remodeling but may contribute to ongoing tissue damage if they remain in a pro-inflammatory state (43) (**Table 1**). Moreover, chemokines regulate T-cell trafficking: regulatory T-cells (Tregs) have been shown to mitigate excessive inflammation and foster repair, whereas effector T-helper subsets such as Th1 and Th17 can potentiate tissue destruction if not properly regulated (48).

**Table 1:** Crosstalk between microglia and surrounding CNS cells after SCI.

Microglia crosstalk after SCI			
*Target cell type*	*Microglia-derived factors/crosstalk signals*	*Engagement & actions after SCI*	*Outcome (beneficial vs detrimental)*
Neurons	• TNF-α, IL-1β, IL-6, ROS/NO • BDNF, purinergic ATP signaling • Neuronal fractalkine (CX3CL1 → CX3CR1 on microglia)	• Microglia contact synapses and axons via specialized junctions. • Clear debris, support survival, influence neurotransmission. • Excess cytokines drive excitotoxicity and synapse loss.	Beneficial: debris clearance, synaptic support, axonal regeneration.
Astrocytes	• Pro-inflammatory: IL-1α, TNF, C1q (→ neurotoxic A1 astrocytes). • Pro-regenerative: IL-6, TGF-β (→ STAT3 activation). • CXCL10 gradients.	• Promote astrocyte proliferation, reactive states, and scar formation. • Without microglia, astrocytes fail to form organized scars, leading to uncontrolled lesion spread.	Beneficial: structured glial scar limits lesion spread, supports repair. Detrimental: excessive A1 astrocytes exacerbate neuronal death and inhibit regeneration.
Oligodendrocytes & OPCs	• Cytotoxic: TNF, NO, complement proteins, S100A8/A9 (→ NF-κB–mediated OPC apoptosis). • Supportive: IGF-1, trophic factors.	• Contribute to demyelination and oligodendrocyte death in early injury. • Microglia also regulate OPC survival and remyelination capacity.	Beneficial: support OPC proliferation and myelin repair. Detrimental: chronic pro-inflammatory activation → demyelination, OPC apoptosis.
Macrophages (infiltrating)	• Microglia release CCL2, CXCL10 to recruit macrophages. • Macrophages release TNF-α, IL-1β (pro-inflammatory) or IL-10, TGF-β (anti-inflammatory).	• Reciprocal amplification of inflammation. • Alternatively, IL-10/TGF-β from macrophages + microglia promote resolution.	Beneficial: anti-inflammatory loop supports repair. Detrimental: pro-inflammatory loop amplifies tissue damage.

Following SCI, resident microglia rapidly activate and engage in complex bidirectional communication with neurons, astrocytes, oligodendrocytes, and infiltrating macrophages.

Therefore, the complete suppression of inflammation is not desirable, as it may impair necessary repair mechanisms such as angiogenesis, neurogenesis, and matrix remodeling.

## 4 Microglia as central responders in SCI

Following SCI, resident microglia rapidly emerge as key regulators of the neuroinflammatory response. Unlike infiltrating macrophages, microglia are intrinsic to the CNS and respond within hours of injury. They engage directly with damaged neurons, axons, and surrounding glial cells, positioning them at the forefront of the injury response (8, 49, 50).

In the early phase post-injury, microglia transition from a surveillant to an activated state in response to damage-associated molecular patterns (DAMPs) and other injury signals (49, 50). This activation is marked by morphological changes, upregulation of pattern recognition receptors, and secretion of pro-inflammatory cytokines. Simultaneously, microglia initiate phagocytosis of cellular debris, which is crucial for limiting secondary injury and initiating tissue repair. This early response not only mitigates damage but also orchestrates the broader immune and glial reactions that follow (1).

Among the glial cells influenced by microglia, astrocytes are particularly responsive (2). Microglia promote astrocyte proliferation and activation by releasing cytokines such as IL-1α, TNF, and complement component 1q (C1q). These signals can drive the formation of neurotoxic astrocytes, which are implicated in exacerbating neuronal injury (35, 51). However, microglia can also support astrocytic functions that are neuroprotective and regenerative, depending on their activation state and surrounding microenvironment (32). Microglia-derived factors such as IL-6, TGF-β activate the STAT3 pathway in astrocytes, which is essential for the development of reactive astrocytes and glial scar formation. In this context, the glial scar is influenced by microglia signals and without them, astrocytes may fail to adopt a reactive, regeneration-supportive phenotype, especially when combined with pro-regenerative factors (52). Importantly, astrocytes are not inherently inhibitory; their function is context-dependent, and largely regulated by microglia activation (**Table 1**).

Supporting this, a study showed that in the absence of microglia, astrocytes become disorganized and reactive in an uncoordinated manner, failing to form an effective lesion boundary. Microglia help establish signaling gradients, including CXCL10 and IL-6, which guide a structured astrocytic response and scar formation. Therefore, the crosstalk between microglia and astrocytes is essential for the development of a functional, protective glial scar that limits lesion spread and supports CNS repair (32).

Microglia also play a significant role in regulating oligodendrocytes and their progenitor cells (OPCs). In the healthy CNS, microglia support myelinogenesis and maintain OPC populations. After SCI, however, demyelination and oligodendrocyte death contribute to secondary injury (53). Activated microglia can exacerbate these effects through the release of cytotoxic mediators such as TNF, nitric oxide (NO), and complement proteins. For example, microglial activation via S100A8/A9 can induce OPC apoptosis through the NF-κβ signaling pathway (**Table 1**). Conversely, interventions like fluoxetine have been shown to reduce oligodendrocyte loss by suppressing harmful microglial activation (54).

Beyond their interactions with glial cells, microglia also engage directly with neurons. They influence synaptic activity and neurotransmission through physical contact, particularly at specialized microglia-neuron junctions involving purinergic signaling (55). These interactions are thought to enhance excitatory transmission and support neuronal survival. Furthermore, direct microglial contact with injured axons may promote regeneration, challenging the conventional perception of microglia solely as agents of inflammation (**Table 1**) (10, 55–57).

From early debris clearance to modulation of astrocytes, oligodendrocytes, and neurons, microglia perform a diverse array of functions that are both beneficial and potentially harmful (5, 58). Importantly, microglial activation is not static, it evolves over time and is highly sensitive to changes in the local microenvironment. While early activation is often neuroprotective, chronic or dysregulated activation can amplify inflammation, contribute to tissue damage, and impair functional recovery (59). The dual role of microglia, as facilitators of repair and drivers of degeneration, highlights their complexity and the need for a better understanding of their function in SCI.

## 5 Microglia: dual roles after CNS injury

The immediate outcome of SCI creates a distinct microenvironment that profoundly influences microglial phenotypes. Previously, microglia have been categorized into two polarized states: M1 (pro-inflammatory) and M2 (anti-inflammatory or reparative) (39, 60). However, recent research challenges this binary classification, suggesting it oversimplifies the complexity of microglial responses. Evidence shows that canonical markers associated with M1 and M2 states are often co-expressed within the same cells, indicating that these phenotypes exist along a continuum rather than as discrete states (61). Instead, microglial activation represents a spectrum of dynamic phenotypes that vary based on spatial and temporal factors, as well as the severity of the injury. Similar complexity is observed in MDMs (62). Collectively, these findings suggest that the conventional M1/M2 context does not adequately capture the complexity of microglial responses after SCI (61).

In the temporal progression, immediately after SCI, the microenvironment is dominated by acute injury signals such as necrotic cells, free radicals, and pro-inflammatory cytokines. Within the first 24 hours post-injury, microglia adopt a predominantly pro-inflammatory phenotype, characterized by peak expression of TNF-α, IL-1β, and IL-6 (43, 63, 64). These cytokines amplify the inflammatory milieu and contribute to blood-spinal cord barrier disruption, leukocyte recruitment, and secondary injury (**Table 1**) (43).

This early phase is crucial, as it sets the stage for subsequent immune responses. As the injury enters the subacute phase (approximately 3 to 7 days post-injury), the intensity of inflammatory cues diminishes, and reparative signals begin to emerge. During this period, microglia display increased phenotypic heterogeneity, with some populations beginning to express anti-inflammatory cytokines such as IL-10 and TGF-β (65). These cytokines modulate inflammation, support tissue repair, and limit further damage by inhibiting key inflammatory pathways such as NF-κβ signaling (63, 64). By seven days post-injury, the reparative profile becomes more pronounced in a subset of microglia, marked by elevated expression of IL-10, TGF-β1, and Arg1 (66). These cells actively participate in clearing apoptotic debris, promoting angiogenesis, and releasing neurotrophic factors like IGF-1 and BDNF, which support neuronal and glial survival (7) and (**Table 2**).

**Table 2:** Cytokine expression by microglia and macrophages after SCI categorized by injury phases.

Cytokine expression by microglia and macrophages after SCI				
*Time post-lesion*	*Pro-inflammatory cytokines*	*Anti-inflammatory cytokines*	*Neurotrophic/repair-associated factors*	*Notes*
Acute phase				
*0–6 hours*	TNF-α, IL-1β, IL-6	—	—	Initiated rapidly via TLR/NLR activation by DAMPs; transcription begins within 30–60 min
*6–24 hours*	TNF-α ↑↑, IL-1β ↑↑, IL-6 ↑↑, IL-12, IL-23, CCL2, CXCL1	Minimal IL-10 (low expression)	—	Blood–spinal cord barrier disruption and leukocyte recruitment; microglia adopt a pro-inflammatory phenotype
Subacute phase				
*3 days*	TNF-α ↑, IL-1β ↑, IL-6 (still elevated), IL-18, CCL2, CXCL10	IL-10 ↑ (beginning), TGF-β1 ↑	—	Beginning of microglial phenotype shift; dual pro- and anti-inflammatory signatures coexist
*7 days*	TNF-α ↓ (but still present), IL-1β ↓, IL-6 ↓, IL-18, iNOS (still elevated)	IL-10 ↑↑, TGF-β1 ↑↑, Arg1 ↑↑, IL-4 ↑, IL-13 ↑ (in some models)	IGF-1 ↑, BDNF ↑, VEGF ↑	Shift to anti-inflammatory-like phenotype; anti-inflammatory & repair-promoting cytokines predominate
Chronic phase				
*≥14 days – weeks*	IL-1β (low but persistent), TNF-α (persistent), IL-6 ↑ (late peak in some models), IL-18	IL-10 (maintained), TGF-β1 (declines but still detectable)	IGF-1, BDNF, GDNF (may fluctuate)	Mixed phenotype (pro- and anti-inflammatory); long-term glial reactivity, scar formation
*≥1 month – chronic*	TNF-α ↑, IL-6 ↑↑ (in severe models), IL-1β ↑	IL-10 ↑ (especially in severe SCI), TGF-β1 ↓ (compared to subacute but still above baseline)	BDNF ↓ (in some models), IGF-1 (variable)	Chronic inflammation and degeneration may predominate; neuroprotective factors may decline

This table summarizes the temporal expression patterns of key cytokines produced by microglia and macrophages following SCI. Expression is grouped by injury phase: acute, subacute, and chronic. The table highlights the dynamic shift from pro-inflammatory to anti-inflammatory and repair-associated cytokine profiles. ↑, Upregulated; ↑↑, Strongly upregulated; ↓, Downregulated compared to the previous time point.

As time progresses into the chronic phase, microglia continue to persist in an activated state but demonstrate a mixed or ambiguous phenotype. While certain microglial populations sustain low-level expression of anti-inflammatory cytokines such as IL-10 and TGF-β (67) to maintain homeostasis and limit chronic inflammation, others may re-express pro-inflammatory markers, contributing to neurodegeneration and glial scarring (**Table 2**).

Importantly, the phenotypic transformation of microglia is not only time-dependent but also spatially heterogeneous. Microglia located at the lesion epicenter are exposed to the most severe damage and inflammatory signals leading to a more intense and sustained pro-inflammatory response. In contrast, microglia in the surrounding, less-damaged parenchyma or in white matter tracts may adopt a different phenotype, focusing on clearing myelin debris or supporting neuronal survival. This spatial diversity allows for a localized, context specific response to injury.

Underlying these temporal and spatial changes are molecular signals originating from damaged cells. Danger-associated molecular patterns (DAMPs), such as high mobility group box 1 (HMGB1) and ATP, are released in abundance following injury and activate microglia (8) through pattern recognition receptors (PRRs), including Toll-like receptors (TLRs) (68), NOD-like receptors (NLRs), RAGE (69), and purinergic receptors like P2X7 (68). These receptor-mediated pathways initiate the production of inflammatory cytokines and the assembly of inflammasomes, such as NLRP3, which further amplify the immune response through the maturation and release of IL-1β and IL-18 (70). At the same time, structural changes in the spinal cord, such as the formation of a glial scar by reactive astrocytes, introduce additional chemical and physical barriers that can inhibit the transition of microglia toward a reparative state. Epigenetic mechanisms, particularly microRNAs (miRNAs), add another layer of regulation (71–73). MicroRNAs such as miR-24-3p, miR-145a-5p, and miR-124-3p modulate microglial gene expression related to cytokine production, survival, and repair, fine-tuning the balance between inflammation and resolution (72).

Finally, the severity of the lesion itself further influences this balance. In cases of more severe SCI, characterized by greater tissue disruption, hemorrhage, and necrosis, the concentration of DAMPs and pro-inflammatory cytokines is markedly elevated and prolonged. This sustained inflammatory environment continuously stimulates microglia and infiltrating macrophages, impeding their ability to transition to reparative phenotypes. In milder injuries, by contrast, the lower burden of inflammatory signals allows for a timelier resolution of inflammation and greater potential for recovery (74, 75). Thus, the microglial response to SCI reflects a complex interplay between when (temporal dynamics), where (spatial distribution), and how much (injury severity). Understanding this multidimensional regulation is essential for developing therapeutic strategies aimed at modulating microglial function to promote repair while minimizing secondary damage.

## 6 Therapeutic strategies

### 6.1 Direct microglial manipulation: reprogramming

New therapeutic approaches for modulating microglia activation after SCI are moving beyond simply suppressing inflammation to focus on reprogramming or guiding microglia towards a beneficial, pro-regenerative phenotype, thereby promoting both neuroprotection and functional recovery. Regulatory T cells (Tregs) have been shown to suppress microglial inflammation by inhibiting STAT3 signaling (76). In SCI models, depletion of Tregs induced a pro-inflammatory microglial state characterized by increased expression of cytokines such as TNF-α, IL-6, IL-1β, and chemokines like CCL2. Conversely, pharmacological inhibition of STAT3 mimicked the anti-inflammatory effects of Tregs, reducing demyelination and improving motor outcomes (76).

CX3CL1 and macrophage colony-stimulating factor (M-CSF) has been shown to promote the centripetal migration of microglia toward lesion cores, displacing infiltrating macrophages. This retention of microglia enhanced phagocytic signaling via SYK kinase and was associated with improved axonal preservation and functional recovery (77) Pharmacological modulation, such as the use of the JAK inhibitor tofacitinib, has also proven effective in reducing microglial inflammation by disrupting the JAK/STAT signaling axis, thereby promoting neuroprotection and motor function restoration in rodent SCI models (78). miR-145a-5p followed by direct transplantation into the injured spinal cord has been shown to boost repair processes and enhance functional recovery (73).

Together, these emerging strategies provide compelling evidence that microglia are not only critical mediators of SCI pathology but also viable therapeutic targets. By leveraging immune, genetic, epigenetic based approaches, it is increasingly feasible to direct microglial behavior away from destructive inflammation and toward regeneration.

### 6.2 iPSC-and pro-regenerative-derived microglia for targeted therapeutic delivery

Engineered microglia derived from induced pluripotent stem cells (iPSCs) have emerged as promising tools for targeted therapy, functioning as “living drug factories” capable of delivering therapeutic molecules directly to sites of neurological injury. Unlike systemic drug administration, iPSC-derived microglia can migrate to injury sites, cross the BBB, and release their therapeutic payloads precisely where needed (79).

Moreover, several studies have demonstrated that exosomes derived from mesenchymal stem cells (MSCs) exert biological effects on target cells by delivering specific microRNAs (miRNAs) (80). A recent study showed that exosomes from hypoxia-preconditioned MSCs promote functional behavioral recovery following SCI in mice through the delivery of miR-216a-5p. This therapeutic effect is mediated by a shift in microglial phenotype from pro-inflammatory to anti-inflammatory, achieved through inhibition of the TLR4/NF-κβ signaling pathway and activation of the PI3K/AKT pathway (81).

In addition to iPSC-derived approaches, direct transplantation of microglia pre-conditioned toward a pro-regenerative phenotype represents another promising therapeutic strategy (82). Preclinical studies have demonstrated that this approach can enhance motor function recovery and upregulate the expression of neuroprotective molecules in CNS (81).

One study employed oxygen–glucose deprivation (OGD) to precondition microglia *in vitro* before transplanting them into the brains of rats subjected to ischemic injury. This intervention led to enhanced axonal outgrowth in the peri-infarct region, as evidenced by increased expression of axonal markers SMI31 and GAP-43, along with a reduction in the inhibitory extracellular matrix component CSPG, suggesting improved neural regeneration (83).

In another study, cultured primary microglia were transplanted into a rat spinal cord injury (SCI) model seven days post-injury. The treatment resulted in significant motor function improvement, as assessed by BBB scores and inclined plate testing from the second week post-transplantation (84). Further, IL-4–induced M2-polarized microglia were genetically engineered to overexpress miR-145a-5p and transplanted into mice following SCI. This combinatorial approach led to a significant enhancement in locomotor function compared to both control and unmodified microglia groups, with higher Basso Mouse Scale (BMS) scores, improved swimming performance, and increased hindlimb reflex scores (73).

Similarly, another study demonstrated that transplantation of IL-4–induced “M2” microglia into a thoracic SCI mouse model resulted in significant recovery of motor function. Compared to M1-treated and control groups, the M2-treated animals showed improved performance on the BMS scale and enhanced hindlimb reflexes, further supporting the reparative potential of pro-regenerative microglia (85).

Collectively, these findings suggest that pre-conditioning microglia toward an anti-inflammatory or pro-repair phenotype, whether through cytokine stimulation, genetic engineering, or metabolic stress, can enhance their therapeutic efficacy following CNS injury.

### 6.3 Targeting specific microglial pathways/receptors

Colony-stimulating factor 1 receptor (CSF1R) signaling is essential for microglial survival and proliferation. Inhibition of CSF1R can selectively deplete pro-inflammatory microglial populations, offering a strategy to “reset” the microglial environment. A study in adult mice demonstrated that administration of the CSF1R inhibitor PLX5622 in chow for 7 days resulted in the depletion of approximately 90% of microglia. Upon withdrawal of the inhibitor, microglia rapidly repopulated the brain, returning to baseline levels. This repopulation phase was associated with reduced inflammatory markers and restoration of synaptic integrity, as indicated by increased PSD95 and synaptophysin expression. Importantly, the treated mice also showed improved functional recovery, supporting the concept that transient CSF1R inhibition removes detrimental microglia and allows for the emergence of a more homeostatic, neuroprotective microglial population that facilitates recovery following brain injury (86).

Microglial depletion by inhibiting CSF1R in models of spinal cord injury (SCI) has demonstrated detrimental effects (5, 32). Microglia are essential for coordinating injury responses, including interactions with CNS-resident glia and infiltrating leukocytes. Their depletion exacerbates tissue damage and impairs functional recovery. However, restoring specific microglia-dependent signaling pathways, identified through transcriptomic analyses, in microglia-depleted mice has been shown to prevent secondary damage and promote neurorepair following SCI (5).

Triggering Receptor Expressed on Myeloid cells 2 (TREM2) is a receptor found on microglia that plays a role in their activation and can contribute to neuroinflammation. Inhibiting TREM2 by using Knockdown of TREM2 reduced phosphorylation of NF-κβ and decreased IL-6 and TNF-α production. This indicates TREM2 drives NF-κβ–mediated microglial activation and inflammation post-SCI (87).

Some novel compounds, such as LRRK2 inhibitors like HT-4253, are being investigated for their potential to suppress innate immune overactivation, particularly in the context SCI. HT-4253 exerts neuroprotective and anti-inflammatory effects by targeting LRRK2, a kinase highly expressed in activated microglia (88). By inhibiting LRRK2 activity, HT-4253 reduces the release of pro-inflammatory cytokines such as TNF-α, IL-1β, and IL-6, and promotes a shift in microglial phenotype from the pro-inflammatory state to the anti-inflammatory state. This modulation of microglial activity helps mitigate secondary injury, preserve neuronal and glial tissue, and maintain white matter integrity. Animal models of SCI were induced, followed by dosing with LRRK2 inhibitors (89). HT-4253 has entered Phase I trials in humans, demonstrating favorable safety and pharmacokinetics (NCT06537817).

Manipulation of the CX3CR1 receptor in microglia has shown significant therapeutic promise in spinal cord injury (SCI) models. CX3CR1-deficient mice (CX3CR1^-^/^-^ or GFP/GFP) demonstrate markedly improved motor function, including higher Basso Mouse Scale (BMS) scores and superior locomotor recovery compared to wild-type controls. These improvements are accompanied by reduced lesion size, enhanced white matter preservation, and greater myelin and axonal integrity (90). The absence or inhibition of CX3CR1 signaling drives microglia and macrophages toward a reparative phenotype, lowering the expression of inflammatory mediators such as iNOS and IL-6 and reducing infiltration of neurotoxic macrophage subsets (e.g., Ly6C^lo^/iNOS^+^), thereby improving the local lesion environment (7, 90). Additionally, CX3CR1 deficiency enhances neuroplasticity by upregulating regenerative gene expression, stimulating NG2 glial activation, increasing serotonergic axonal sprouting, and promoting synaptogenesis in motor circuits. At the lumbar level, this corresponds with reduced dendritic degeneration and stronger synaptic connectivity in ventral motor neurons (7). Interestingly, CX3CL1 (fractalkine) signaling via CX3CR1 also activates NF-κβ in microglia, which paradoxically restrains excessive inflammatory responses through downregulation of Iκβ and P65 phosphorylation, contributing to neuronal protection (91). Collectively, these findings highlight CX3CR1 as a key immunomodulatory target with multifaceted benefits for SCI repair, from controlling inflammation to enhancing structural and functional recovery.

### 6.4 Pharmacological modulators

The pathophysiology of SCI is dominated by a cascade of secondary injury processes, in which microglia-driven inflammatory and excitotoxic mechanisms plays a central role. Pharmacological interventions targeting these inflammatory and excitotoxic mechanisms have therefore been investigated as therapeutic strategies to limit secondary damage and promote functional recovery.

Methylprednisolone (MP), a synthetic corticosteroid, was one of the first agents tested for SCI. Its primary mechanism is to suppress pro-inflammatory cytokines by activating the glucocorticoid receptor. When given immediately after injury, MP has been shown to decrease TNF-α expression by approximately 50% and inhibit the action of NF-kβ, a transcription factor that drives the production of downstream cytokines and chemokines (92). Studies in injured spinal cord models have also shown that MP reduces the extracellular release of excitatory amino acids (e.g., glutamate) and limits the cascade of ionic dysfunction, which can lead to secondary neuronal death. By reducing excitotoxic burden and oxidative injury, MP indirectly lessens the stimuli that sustain microglial activation (93). Recent studies show that MP attenuates microglial and astrocyte pro-inflammatory phenotypes, reduces caspase activation and apoptosis, and improves histological spacing and locomotor measures when administered early at appropriate doses (94).

Minocycline, a tetracycline derivative, is known for its potent anti-inflammatory and anti-apoptotic properties, which go beyond its typical antimicrobial activity. This drug inhibits microglial activation, suppresses matrix metalloproteinases, and reduces caspase-dependent cell death. In rodent models, minocycline consistently reduced lesion size, preserved white matter, and improved motor skills (95–98). Interestingly, minocycline’s effectiveness is highly dependent on when it’s administered. In rats with spinal cord injuries (SCI), giving minocycline one hour after the injury significantly reduced lesion size, inhibited mitochondrial cytochrome C release, and improved motor recovery. However, delayed administration (over 24 hours) lost most of these benefits (96, 98). Similarly, administering the drug earlier (less than 12 hours) also reduced caspase activation, microglial reactivity, and improved tissue preservation, but these neuroprotective effects were significantly reduced when the treatment was delayed (97).

Riluzole, a sodium channel blocker originally developed for amyotrophic lateral sclerosis (ALS), reduces persistent sodium influx and thereby limits glutamate release and excitotoxicity. By mitigating excitotoxic stress, riluzole indirectly reduces microglial activation and neuronal death. Pre-clinical studies demonstrated robust neuroprotective effects, leading to the Riluzole in Spinal Cord Injury Study (RISCIS), a large international Phase III trial. Riluzole was associated with improved global outcomes in patients with severe traumatic SCI, based on a composite score integrating ASIA total motor scores, SCIM, and SF36 outcomes. Additionally, the trial confirmed riluzole’s safety and revealed positive signals in prespecified subgroup and secondary analyses, suggesting potential benefit in selected patient populations (99–101).

Gabapentin, widely used for neuropathic pain and spasticity in spinal cord injury (SCI), may indirectly affect microglial activity by modulating calcium channels. The drug also reduces inflammation by acting on key molecular pathways. Studies show it can decrease pro-inflammatory cytokines like IL-1β, TNF-α, and IL-6, and inhibit the activity of the transcription factor NF-κB, which regulates inflammatory responses (102).

A study found that giving gabapentin to mice early after SCI prevented damaging structural changes in their spinal cords. This suggests gabapentin could be a prophylactic therapy to prevent the development of autonomic dysfunction, a serious SCI complication that can lead to high blood pressure and immune suppression (103). A clinical trial (NCT05302999) is currently underway to assess if early administration of gabapentin can specifically aid neurorecovery, not just pain management.

Another drug, GW2580, a CSF1R inhibitor, has demonstrated potential for reducing microglial proliferation and neuroinflammation while enhancing functional outcomes in models of neurodegenerative diseases (104). In SCI models, oral administration of GW2580 has been shown to suppress microglial proliferation. In studies involving mice and nonhuman primates, treatment with GW2580 improved locomotor function and reduced spinal cord pathology following injury (105). Furthermore, treatment initiated at chronic stages, specially at 42- and 84-days post-injury, led to reduced microglial proliferation and altered morphology, indicating potential modulation in chronic SCI (106).

Other anti-inflammatory and neuroprotective drugs have also been explored. Non-steroidal anti-inflammatory drugs (NSAIDs), such as ibuprofen, inhibit cyclooxygenase-mediated prostaglandin synthesis and modulate RhoA signaling, a pathway that inhibits axonal regeneration. Pre-clinical studies showed that ibuprofen reduced microglial activation and promoted axonal growth, though no large clinical trials have confirmed benefit in SCI (107). Progesterone, a neurosteroid with potent anti-edema, anti-apoptotic, and anti-inflammatory effects, improved locomotor function and reduced gliosis in rodent SCI models (108).

Additional agents with anti-inflammatory or antioxidant properties have also been studied. Statins, particularly simvastatin and atorvastatin, inhibit NF-κβ signaling, suppress microglial activation, and reduce pro-inflammatory cytokine release. Pre-clinical studies demonstrated functional improvements (109, 110) and small clinical trials suggested possible benefit, but evidence remains preliminary.

Taken together, these pharmacological strategies demonstrate that targeting neuroinflammation and secondary injury processes remains a promising avenue for SCI treatment.

### 6.5 Controlling BBB disruption to enable repair

Disruption of the blood–brain barrier (BBB) is a hallmark of SCI and contributes significantly to the progression of secondary injury. Damage to the BBB permits the infiltration of peripheral immune cells, pro-inflammatory cytokines, and neurotoxic plasma proteins into the CNS parenchyma. This infiltration exacerbates microglial and astrocytic activation, triggering a self-amplifying cycle of neuroinflammation that promotes neuronal death and impedes tissue repair (111). Therefore, understanding and targeting the interplay between BBB dysfunction and innate immune activation is critical for developing strategies to contain inflammation and facilitate neural recovery after SCI.

Among the key cellular players at the neurovascular interface are brain pericytes, which help regulate leukocyte trafficking across the BBB (112, 113). Upon stimulation by inflammatory mediators such as TNF-α, IL-1β, and bacterial endotoxins like LPS, pericytes increase the production of IL-8 and matrix metalloproteinase-9 (MMP-9). These molecules work synergistically to facilitate neutrophil migration into CNS tissue (112). *In vitro* coculture studies have shown that blocking IL-8 signaling reduces neutrophil migration, while MMP-9 inhibition prevents neutrophil detachment from pericytes, underscoring MMP-9’s role in promoting trans endothelial migration. Thus, inflammatory pericytes directly contribute to barrier compromise and leukocyte infiltration by modulating IL-8 and MMP-9 signaling, suggesting that targeting these pathways may mitigate BBB disruption and its downstream effects (114).

Pharmacological agents such as minocycline, statins, and VEGF inhibitors have shown considerable potential in preserving BBB integrity and mitigating inflammatory damage. Minocycline, a tetracycline derivative with broad anti-inflammatory and neuroprotective effects, has been demonstrated to inhibit microglial activation, reduce pro-inflammatory cytokine production (e.g., TNF-α, IL-1β), and downregulate MMP-9 activity. This suppression of MMP-mediated tight junction degradation preserves endothelial integrity, reducing vascular permeability and limiting leukocyte extravasation (115). Similarly, statins exert protective effects through enhancement of endothelial nitric oxide synthase (eNOS), inhibition of adhesion molecule expression, and suppression of NF-κβ signaling (110). VEGF inhibitors, while traditionally used in oncology and ophthalmology, are being repurposed to counteract VEGF-A–induced vascular permeability in the acute phase of SCI, thereby reducing edema and barrier breakdown (116).

**Figure 3:**
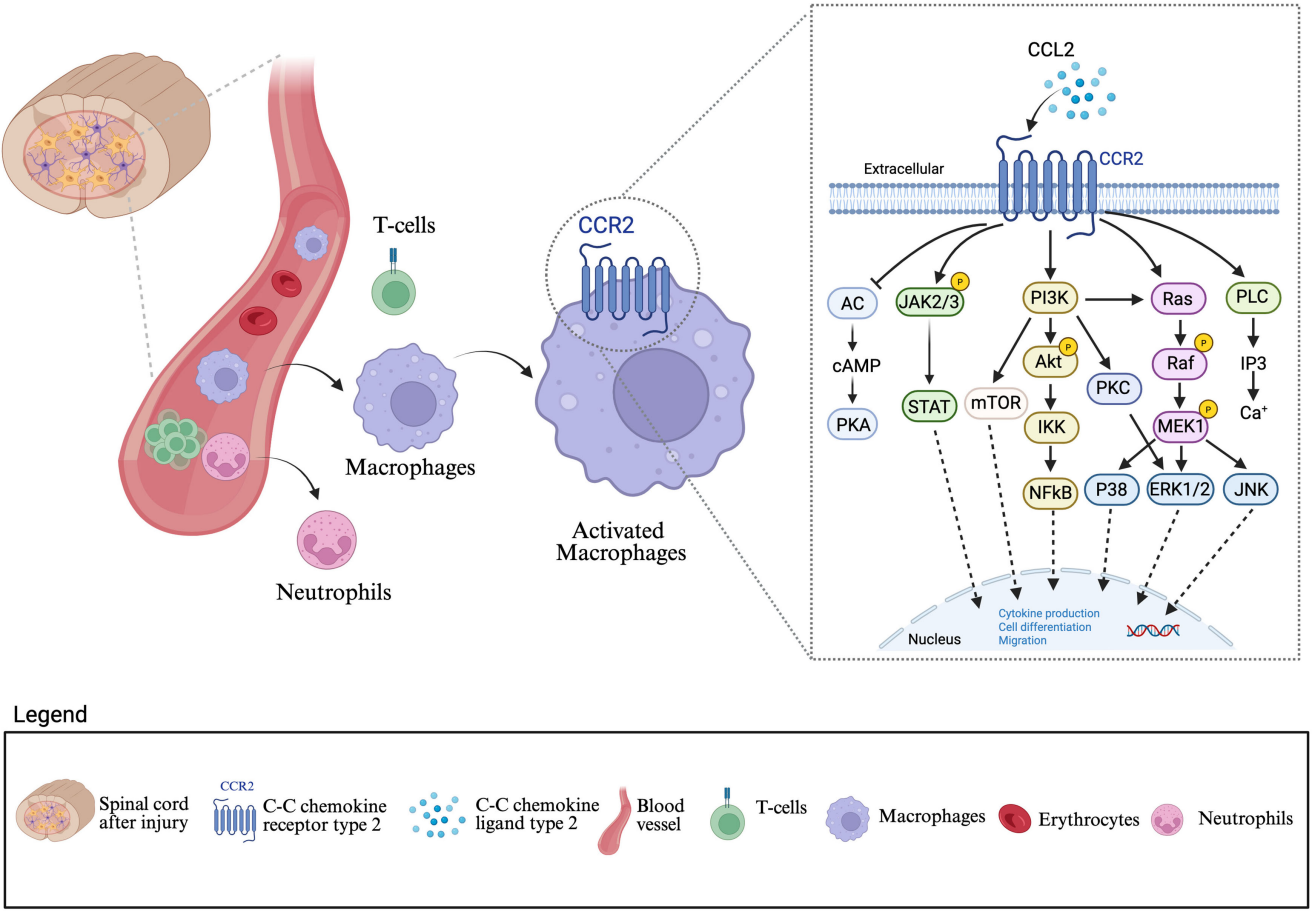
Blood brain barrier disruption and immune cell infiltration mediated by the CCL2–CCR2 axis after spinal cord injury: Following a spinal cord lesion, disruption of the blood brain barrier (BBB) permits infiltration of peripheral cells, including neutrophils, T cell, and macrophages into the central nervous system (CNS) parenchyma. Among these cells, macrophages play a central role in amplifying neuroinflammation through the CCL2–CCR2 signaling pathway. CCL2, secreted in response to injury, binds to its receptor CCR2 on macrophages, leading to their activation. Activated macrophages initiate downstream signaling cascades involving JAK/STAT, PI3K/Akt/mTOR, Ras/Raf/MEK/ERK, and PLC/PKC pathways. These pathways converge to regulate nuclear transcription factors such as NF-κβ, STAT, and AP-1, which promote cytokine production, cell differentiation, and migration. Collectively, this signaling axis drives sustained macrophage recruitment and activation, exacerbating secondary injury and chronic neuroinflammation after SCI. This figure was created with BioRender.com.

Complementing these strategies, modulation of chemokine signaling pathways such as the CCL2–CCR2 axis provides another promising avenue for intervention. CCL2 (MCP-1), rapidly upregulated after SCI by activated astrocytes, microglia, and endothelial cells, acts as a potent chemoattractant for CCR2-expressing monocytes (47). The resulting influx of peripheral macrophages into the spinal cord parenchyma amplifies inflammation and contributes to further BBB breakdown (**Figure 3**). Inhibition of CCR2, either genetically or pharmacologically, has been shown to reduce monocyte-derived macrophage infiltration, diminish pro-inflammatory cytokine production, and preserve barrier function (117, 118). Animal models treated with CCR2 antagonists or CCR2 knockout exhibit reduced lesion volumes, decreased BBB leakage, and improved functional recovery. Additionally, blocking CCL2–CCR2 signaling shifts macrophage populations away from the pro-inflammatory Ly6C^hi^ phenotype toward a more reparative, anti-inflammatory state, fostering a more favorable environment for neuroregeneration (117).

Collectively, these findings highlight the importance of protecting BBB integrity not only to limit immune cell infiltration and inflammatory damage but also to modulate microglial phenotype. A combination of therapies, targeting pericyte activity, MMPs, and chemokine signaling, offers a multipronged approach to stabilize the neurovascular unit, reduce secondary injury, and promote meaningful repair after SCI.

## 7 Clinical trial challenges & insights

Despite the promise of microglia-targeted therapies for spinal cord injury (SCI), translating preclinical success into effective clinical interventions remains a significant challenge. Numerous preclinical and clinical trials have focused on modulating the inflammatory cascade after SCI, using approaches such as immune-modulating drugs, cell-based therapies, exosome-based strategies, and anti-inflammatory diets (**Table 3**). Findings from these studies highlight key translational barriers and suggest directions for future development.

**Table 3:** Pre-clinical and clinical trials for SCI anti-inflammatory treatments.

Summary of pre-clinical and clinical trials for SCI anti-inflammatory treatments			
*Treatment*	*Lesion stage & number of patients*	*Functional outcomes (Results)*	*Success/failure analysis*
Methylprednisolone (NASCIS Trials, multiple papers)	Acute: 48–72 hours post-injury. NASCIS 2: 487 patients. NASCIS 3: 499 patients.	• NASCIS 2: Modest improvement in motor and sensory function if treated within 8 hours. • NASCIS 3: 48-hour course better than 24-hour for patients treated 3–8 hrs post-injury.	Controversial Success • Benefits: Some neurological improvement. • Failures: High risk of severe side effects (sepsis, GI bleeding); not routinely recommended anymore.
Minocycline (Multiple Phase II studies)	Acute: Within 12 hours post-injury. Phase II trials: ~25–50 patients per group.	• Trend toward improved motor recovery, especially in cervical SCI. • Did not reach statistical significance.	Failed to Show Efficacy • Benefits: Safe and well-tolerated. • Failures: No significant functional benefit; not advanced to Phase III.
Riluzole (RISCIS trial)	Acute: Within 12 hours post-injury. ~130+ patients.	• Significant motor improvement at 6 months and 1 year, especially cervical SCI. • Safe with minimal side effects.	Promising Success • Benefits: First drug to show clear benefit using Global Statistical Test. • Failures: Not a cure; gains were functional, not full recovery.
Gabapentin (Literature in SCI pain/spasticity)	Subacute to chronic. Clinical trial (NCT05302999)	• Improvement in neuropathic pain, spasticity, and autonomic symptoms. • May indirectly modulate microglial excitability.	Supportive Adjunct • Benefits: Safe, widely used. • Failures: No SCI-specific; mechanistic effect on microglia not fully validated in humans.
CSF1R Inhibitors (e.g., PLX5622, GW2580)	Preclinical only (rodent models)	• Reduced microglial proliferation and neuroinflammation. • Improved functional recovery in SCI mice.	Preclinical Promise • Benefits: Highly specific microglia modulation. • Failures: No human trials yet; dosing and long-term effects unknown.
Cell-Based Therapies (varied stem/progenitor cells trials)	Acute & Chronic. Varied: trials and instead and case series (5–10 patients/early phase)	• Variable improvements in AIS grade, motor/sensory scores.	Emerging Success • Benefits: Anti-inflammatory + regenerative effects.
Anti-inflammatory Diet (NCT02099890)	Chronic SCI with neuropathic pain; small trials (~20 patients).	• Reduced neuropathic pain and pro-inflammatory markers (IL-1β, IL-6, IFN-γ, PGE_2_).	Modest Success • Benefits: Safe, non-pharmacological; symptom improvement. • Failures: Small study; unclear long-term impact on function.

This table summarizes clinical and translational studies investigating anti-inflammatory and microglia-modulating treatments for SCI. Treatments are grouped based on their stage of clinical development (acute, subacute, chronic, or preclinical) and include pharmacologic agents, cell-based therapies, dietary interventions, and investigational microglial modulators.

Methylprednisolone, once considered a standard therapy, is no longer routinely recommended. Although preclinical studies consistently demonstrated neuroprotective and anti-inflammatory effects, clinical trials reported only modest motor improvements when the drug was administered within eight hours of injury. Subsequent meta-analyses raised concerns about methodological limitations, limited efficacy, and high rates of complications, including infections and gastrointestinal bleeding (119, 120).

Minocycline has shown encouraging results in Phase II randomized controlled trials with acute SCI patients, where treatment was associated with improved motor recovery and acceptable safety. Cerebrospinal fluid (CSF) analyses revealed reduced levels of inflammatory mediators. Although trends toward motor recovery were observed, particularly in cervical SCI, statistical significance was not reached (121, 122). Consequently, despite being well tolerated, minocycline has not advanced to Phase III trials due to inconsistent efficacy.

One of the obstacles for drug treatments is the timing of therapeutic delivery. The neuroinflammatory response after SCI is highly dynamic, with microglial phenotypes rapidly shifting between protective and harmful states. Preclinical studies with both minocycline (96, 98) and methylprednisolone (94) indicate that earlier treatment tends to yield better outcomes. However, intervention too soon after injury may interfere with necessary debris clearance, while delayed treatment risks missing the critical window for influencing microglial behavior effectively (6, 33).

Other compounds, such as riluzole, have also demonstrated potential. In patients with severe cervical SCI (AIS A, B, and C), treatment initiated within 12 hours of injury and continued for 14 days resulted in significant neurological and functional improvements. Notably, patients with complete injuries (AIS A) and those with partial preservation of motor function (AIS B and C) showed the greatest benefit (100). While riluzole is not a universal solution, these results suggest it could be particularly valuable for specific subgroups of patients with severe injuries.

The varying responses to treatment among patients, known as patient heterogeneity, is a major factor influencing trial outcomes. A drug may be highly effective in a particular group, such as those with a specific injury severity or who are treated within a narrow time frame, but fail to demonstrate a benefit when the study population is too broad. Therefore, identifying and focusing on these specific, responsive subgroups is crucial.

Limited number of participants can be an obstacle to achieving definitive results, even when preliminary findings are positive. A pilot trial (NCT02099890) of an anti-inflammatory dietary intervention for chronic SCI patients with neuropathic pain yielded promising results, showing a reduction in inflammatory markers (IL-1β, IL-6, IFN-γ, and PGE_2_) and improved pain scores (123). However, the trial’s small sample size limited the ability to draw broader conclusions.

Another challenge is translating successful preclinical findings from animal models to human trials. For instance, CSFR1 and CX3CR1 inhibitors, which target microglia, have shown beneficial effects in rodents, reducing inflammation and improving functional recovery. The success of drugs like GW2580, a CSF1R inhibitor, is highly dependent on timing of administration (105, 106). While these preclinical results are encouraging, no human trials have been started yet. Further research is necessary to fully address critical factors such as safety, optimal dosing, and the ideal timing to ensure the drug’s effectiveness can be sustained in humans.

The challenges in translation are compounded by the lack of reliable biomarkers to distinguish between microglial subtypes or to monitor the neuroimmune environment in real time. This impedes accurate patient stratification and the ability to tailor treatments. Further complicating translation is the complexity of microglial signaling pathways, such as JAK/STAT, NF-κβ, CSF1R, TREM2, and CX3CR1, which exert context-dependent effects that are not yet fully understood (124, 125). While pharmacological agents like minocycline, JAK inhibitors, and LRRK2 inhibitors have demonstrated beneficial effects in preclinical models, their broad activity raises concerns about off-target effects and unintended immune suppression (115, 126).

Multi-target approaches aiming to modulate inflammatory cascade following SCI are likely necessary to achieve meaningful recovery in clinical trial. Beyond microglial modulation, preserving the blood brain barrier and controlling peripheral immune cell infiltration, such as through CCR2 antagonism (39, 90, 127), appear essential. Despite strong pre-clinical evidence for targeting the CCL2-CCR2 axis, no clinical trials have yet explored how to combine microglial modulation with strategies to enhance BBB integrity in SCI patients. Advancing translation will require precision medicine approaches tailored to both immune responses and barrier dynamics.

In parallel with pharmacological approaches, novel therapeutic avenues are emerging. Exosomes, tiny vesicles released by cells, are being explored for their ability to carry signaling molecules and promote intercellular communication. Exosomes derived from mesenchymal stem cells (MSCs) have shown promise in promoting tissue repair and modulating inflammation in SCI models (128, 129). Similarly, innovative strategies involving iPSC-derived or pre-conditioned pro-regenerative microglia offer high specificity in targeting injury responses (130, 131). While preclinical studies on these strategies are innovative, there are currently no registered clinical trials specifically for iPSC-derived microglia for transplantation or therapy. However, an ongoing clinical trial in Japan is using iPSC-derived neural progenitor cells (NPCs) for SCI. The study’s primary goal was to assess the safety and effectiveness of transplanting these cells into the spinal cord to promote nerve regeneration and functional recovery. The proposed mechanism of action for this therapy may involve two key processes, which was remodeling of the injury environment through secrete trophic factors and suppression of inflammation in the injury site (132). Although early clinical reports suggest improved motor and sensory function in some patients receiving cell-based therapies, large-scale clinical trials are still lacking. Cell-base therapies face practical barriers, including complex production protocols, scalability issues, and regulatory obstacles. Greater research is needed to identify optimal cell types, transplantation timing, dosage, and potential synergies with other therapies (133).

Ultimately, future therapeutic strategies must embrace the complexity of microglial biology. Personalized, multi-targeted interventions guided by biomarker profiling offer the greatest potential to harness microglial plasticity for neurorepair, while minimizing the risks of chronic inflammation or immunosuppression.

## 8 Conclusion

Spinal cord injury (SCI) triggers a complex neuroinflammatory response where microglia, the resident immune cells of the CNS, play a central role in both inflammation and repair. Initially, their activation is crucial for limiting damage, clearing cellular debris, and initiating the repair process. However, this response is highly dynamic and can shift from protective to detrimental over time. In the acute phase, microglial activation aids in containing the injury and preventing further harm, but prolonged or dysregulated activation leads to excessive release of pro-inflammatory and cytotoxic mediators, which can exacerbate secondary injury and impair regeneration.

The dual nature of microglial function, protective in the short term but potentially harmful in the long term, poses significant challenges in developing effective therapeutic strategies for SCI. Microglia, which under normal conditions contribute to brain plasticity and immune defense, can, in the setting of SCI, become agents of neurodegeneration. This underscores the importance of understanding the mechanisms that govern microglial activation and polarization at different stages of injury and repair.

While several promising therapies targeting microglia reactivity have been explored, such as CSF1R inhibitors, CCR2 antagonism, cytokine modulation via pharmacological agents, microRNAs and stem cell-based approaches, achieving a delicate balance between pro-inflammatory and anti-inflammatory responses remains critical. A major challenge lies in modulating microglial polarization to resolve harmful inflammation while promoting regenerative processes is a major challenge. The key to advancing SCI treatment lies in developing precision-based, multi-targeted interventions that can modulate the microglial response in a way that supports repair and minimizes secondary damage. These interventions must also preserve the blood-brain barrier and limit peripheral immune infiltration.

Despite the promising preclinical data, clinical translation is complicated by the dynamic nature of microglial function and the lack of reliable biomarkers to monitor these responses in real-time. This, combined with narrow therapeutic windows, and patient-specific variability often leads to inconsistent or modest clinical outcomes. Ongoing research into microglial biology, along with insights from animal models and early-stage clinical trials, is moving us closer to understanding how to manipulate these cells to achieve better outcomes for SCI patients.

Ultimately, the future of SCI therapies will depend on harnessing the full regenerative potential of microglia, guided by a deeper understanding of their dynamic roles in neurorepair. Interventions to address the narrow therapeutic windows and patient-specific variability, such as using biomarker-based patient stratification, present the most promising strategy for substantial recovery after SCI.
